# South Texas Medical Association

**Published:** 1899-07

**Authors:** 


### South Texas Medical Association.

The semi-annual meeting of the South Texas Medical Association was held in Cuero, Texas, June 13, 14 (ult.) This is a strong organization with a good membership, and the members take a deep interest in its meetings. Dr. H. A. West, the well known ex-professor of medicine in the Texas Medical College, is president, and Dr. O. S. Hodges, of Galveston, is secretary. We are indebted to Vice-President Dr. Jos. H. Reuss, of Cuero, for the following outline of the proceedings, taken from the *"Cuero Star,"* and to the *"Cuero Record"* for the "Recollections of the Banquet."

The meeting was called to order by Dr. J. H. Reuss, Vice-President of the Association, in a few appropriate words to the profession. Dr. E. W. Woodson, President of the DeWitt County Medical Society, and R. A. Pleasants, Esq., in behalf of Mayor Woodworth, welcomed the M. D.'s in cordial, appropriate terms. The response was made by Dr. H. A. West, of Galveston, President of the Association.

Routine work of calling the roll, reading the minutes, collecting dues, etc., was the next in order, and the material affairs of the society were found to be in excellent condition.

The professional part of the meeting proper was taken up in a discussion of yellow fever and quarantine by Dr. H. A. West. The doctor's remarks were devoted largely to preventing unnecessary and injurious quarantines. Quarantines for the past two or three years he argued could have been avoided; isolation and disinfection, he thought would have met every emergency in these scares. He mentioned the act of the board of health of Mississippi in having a representative at New Orleans to watch all developments of yellow fever and avoid senseless quarantines or possible deception by the board of health, and said that Texas and other Southern States might also adopt this safeguard. The doctor viewed with satisfaction the wholesale cleaning up and renovation the United States is doing in Santiago, Havana and other hotbeds of fever in Cuba, as tending to lessen the liability to the contagion.

The doctor was in favor of a State board of health instead of leaving all the labor and responsibility of guarding the health of the State to one man. It was impossible, he thought, to cover so much territory and to thoroughly protect our public health under the present system.

The venerable Dr. J. M. Reuss, the dean of the Association, came next with a lengthy and highly instructive paper on yellow fever, giving copious reviews from his experience with the fever in 1852, 1867, and intermediate attacks at old Indianola. There was so much historical interest concerning those awful epidemics that we hope at an early date to give the paper, or at least to give free quotations from it. The doctor's paper was very heartily appreciated by the Association as showing far more than usual experience and research with the awful malady. Dr. Reuss spoke of the mild forms of the fever during the past year or so, but thought we had no assurance that its next appearance might not be of very virulent form. Drs. Scott and Morris of Houston took up the discussion. Dr. Scott referred to the fact that yellow fever scares of late years had cost some of our trade centers ten million dollars. Dr. Morris believed that the yellow fever outlook was greatly improving and that in its possible return it would likely be of mild character. During his argument he brought out the fact probably not generally known to the laity that Philadelphia had at times experienced the most aggravated forms of yellow fever ever seen in this country. Dr. Shropshire, of Yoakum, did not take so roseate a view of the situation and believed that quarantines were principally to save life and stamp out the disease, and he did not believe any financial consideration should induce health officers to relax necessary precautions and safeguards.

Dr. Mullen, of Houston, read a very able and comprehensive paper on diphtheria and kindred complaints. He advocated strongly antitoxin, with intubation in some cases, and tracheotomy only when the other remedies do not avail. At night this subject was continued and a very able paper was presented by Dr. D. B. Blake of this city. The discussion then took a wide range on this timely subject. Both gentlemen have had a wide and successful experience with this dread disease and their papers were received with the keenest interest. Dr. Mullen is a specialist, devoting his time exclusively to throat affections and kindred branches, and Dr. Blake's study of the subject and its later management has been clear and thorough and his treatment of it strikingly successful.

During the morning session, at the Knights of Pythias building, a number of very able papers were presented on strictly scientific subjects.

The papers and discussions were largely technical and of more interest to the profession than to laymen. Among some of the papers presented were by Drs. Crouse, of Victoria; Knox, Scott and Morris, Houston; McDaniel and Hadra, San Antonio.

In the afternoon Dr. Shropshire, of Yoakum, read a paper on heart affection, and Dr. E. W. Woodson read an elaborate argument in favor of high education for physicians, which was very warmly discussed and pretty generally endorsed. The doctor contended that about eleven years should be devoted to the literary and professional education of the physician. The idea of thorough equipment seemed to meet the views of the physicians present with striking unanimity.

Dr. Knox's subject was an interesting paper on the surgical treatment of an irritable bladder.

Dr. Crouse considered two different methods of doing vaginal hysterectomy, inclining to Prior's as safest for the patient. The paper was heartily commended.

Dr. Alfred C. McDaniel, of San Antonio, argued for conservatism in pelvic and abdominal surgery, showing acute cases thus cured that would have died under radical operations. His paper was very warmly discussed.

Dr. Hadra, of San Antonio, exhibited strange instances of intestinal disorders, showing that such could arise from swallowing watermelon seed, grape seed, fish bones and the like, and that such could foster fevers and diseases. [Published herewith.—Ed.]

"How long should parturient women remain in bed," was the paper of Dr. J. W. Scott, of Houston, and it brought out some able discussions, but the prevailing view seemed to be that this was largely governed by the nature of the case and the constitution of the individual.

The feature of the paper of Dr. Walter Shropshire, of Yoakum, on "Acute Endocarditis as an Idiopathic Disease," or heart affection without primary cause, was the large number of such cases that had fallen to his lot in the practice. It was highly interesting.

Dr. R. T. Morris, of Houston, did not give the paper regularly assigned him on the program, but took up a line of abdominal disorder and treated it very intelligently and to the edification and satisfaction of the assemblage.

Dr. J. H. Reuss had a very able paper on a branch of abdominal surgery, citing three cases well known here, he has treated, in which results had been very gratifying. Dr. Reuss' paper was one of the ablest contributions in the surgical line and was received with the liveliest interest and appreciation by the Association. "Hystero-

Epilepsy, with a report of three cases entirely relieved by a double Ovariotomy," was the technical title of the doctor's paper.

Dr. W. S. Swain, of Victoria, closed the papers in a very interesting discussion on a case of fever, which was very generally discussed by the meeting.

EXECUTIVE SESSION.

A judicial council for the occasion was appointed by the chair and consisted of J. W. Scott, J. H. Reuss and A. A. Brown, who passed on and recommended the acceptance of some ten applicants, which was done by vote. In the future acquisition of members it was decided to admit them merely on a certificate of good standing with county associations.

By vote it was decided to divide the papers for the December meeting at Houston into four sections in charge of the following chairmen, respectively: Walter Shropshire, of Yoakum, general medicine; J. H. Reuss, Cuero, gynecology or diseases of women; H. W. Crouse, Victoria, general surgery; J. H. Mullen, Houston, diseases of eye, ear, nose and throat.

The doctors are very deeply appreciative of the concert rendered Tuesday night, which was truly a magnificent tribute to them.

The following physicians have been in attendance:

Drs. J. M. Thompson and W. H. Shimer, Meyersville; W. T. Thornton, H. W. Crouse, W. S. Swain, F. B. Shields, Victoria; T. H. Nott, Goliad; J. A. Yates, Hanover; C. A. Arnecke, Arneckeville; W. Westervelt, Corpus Christi; H. A. West and O. S. Hodges, Galveston; J. E. Pridgen, Thomaston; H. H. Brown, Yorktown; Alex Irvin and A. A. Brown, Wallis; D. B. Blake, Cuero; John C. Blake, Floresville; C. B. Phillips, E. W. Woodson, Cuero; E. A. Malsch, Edna; J. M. and J. H. Reuss, Cuero; Joseph Mullen, Houston; J. H. Burleson, Cuero; J. M. Lackey, Cuero.

The meeting throughout was a grand one both for the doctors and for those of our citizens who have availed themselves of the privilege of attending the sessions.

At the adjournment the Association pased resolutions cordially thanking the ladies of the city, the DeWitt County Medical Society and citizens of the city for their marked hospitality.

THE DOCTOR'S BANQUET.—WHAT AN OBSERVER HAS TO SAY ABOUT THE MEDICO'S RECENT MEETING.

Aside from the music, the flowers, the viands and the liquid refreshments, the banquet to the doctors on Wednesday night was a most entertaining and instructive occasion from a literary standpoint.

There were no prepared speeches—every response was impromptu, the respondent in each instance being wholly unaware of the part he was to perform until a few minutes before hand.

In the midst of the feast Dr. J. H. Reuss arose and spoke of the occasion, its purposes, aims and accomplishments. He spoke in most complimentary terms of the visiting members, their earnestness, social and moral worth and enthusiasm. Referring to the profession he said: "Its nature, its scope and its history, as well as its hopes and future, remove all egotism from the expression when I say that I feel it to be the greatest of all the vocations of men." He then adverted to the social side of the physician's life, and said: "The very character of our profession is such that we have little opportunity for frequent association with other than our brother physicians. This is mainly due to the irregularity and uncertainty of our going and coming—it is also due in part to the congeniality necessarily existing between minds which are exploring the same fields. Our leisure is too often the time of occupation of others, and our contact with others most frequent at times when distracting thoughts concerning the well-being of loved ones exist." He expressed the delight of physicians to mingle with men in other vocations, and said that the occasion, while accidental, was one at which he hoped an exchange of thoughts and sentiments would result not only in entertainment, but also in a feeling of nearer relationship between various business and occupations. In concluding his remarks he named Hon. A. B. Davidson toastmaster for the evening, and proposed that the toast to which he should respond be "Our Guests."

Mr. Davidson's address was characteristic. In well-chosen terms he congratulated the Association and praised its purposes, and said that he expressed the opinion of the city when he stated that the personnel of the guests was the occasion of pride to all, and that it was the desire of all that they be entertained in a manner due them. He said that SIXTEEN PEOPLE now living would die after having lived part of three centuries, the eighteenth, nineteenth and twentieth; that those persons had seen the advent of many discoveries, inventions and changes, resulting in benefit and advantage to the human race, in all manners affecting it, from the methods of government, war and commerce to the methods applicable to individual affairs, in the performance of labor and healing of disease, citing numerous illustrations. He spoke of legislation as affecting the privilege of business as well as men, and affecting the health of the people, and concluded his remarks proposing the toast "Our Profession," naming Dr. Crouse, of Victoria.

Dr. Crouse is a most affable and pleasing gentleman, of attractive, dignified bearing. He is possessed of much general information and shows upon his face that he possesses a keen perceptive faculty, as well as caution. He apologized to those present, not physicians, for the fact that he felt constrained to laud his profession, but thought in doing so he detracted naught from their vocations. He spoke of the confidential relationship between the physician and patient, the important confidence reposed, to violate which would often result most disastrously, and said that it was the occasion of much pride of the profession that so few instances of violated confidences had occurred among them. He closed his remarks by reading a toast to wine, which was faultless in construction, chaste language and beautiful in sentiment, the expression, voice and elocution of the reader emphasizing the beauty of the production.

Hon. Davidson said he was going to introduce a man who knew something about everything, or he believed that he did, and that if he did not it would be hard to make that man admit it; he said: "That man you all know; his toast is 'Universal Knowledge;' his name is Judge S. F. Grimes." Knowing the friendship between the toastmaster and "that man," the home people knew (and visitors correctly surmised) there was fun ahead. The judge said if he knew exactly what the symptoms were he would couclude that he had had a slight attack of mental paresis when he heard his name was coupled with the toast proposed. He said that he had not lived through three centuries but had seen the better part of one, and from what he had read and seen, was constrained to believe that all the great and good things do not belong to this day and time, and that especially was this true as to legislation; that in the good old days men lived and enjoyed life because there was competition between individuals, and he thought that Bro. Davidson ought to be the last man to forget the existence of trusts. He said that he thought that it was a mistake to bring a fossil like himself before a body of men as enthusiastic over the present as those present to talk of new things. "I am attached to the past—most of me is in the past; I have a present and not much future. The wars of today are not as deadly as when men locked shields and fought with a short sword, nor are the engagements of ironclad ships with thirteen-inch guns any more effective than the engagements of wooden ships with small bore guns. Decatur and Paul Jones will live as long as Dewey and Schley." The judge conceded that many changes and improvements had been made in medicine and in surgery, but that as far as results were concerned it is now like it was long ago, they then cut and carved with knives that were not "sterilized," and some died and some got well just as they do now—and he added significantly, "the undertaker is still doing business." The judge was in fine trim and made a splendid speech; he created much laughter by his sarcasm and humor—more than scoring the toastmaster, and pleasantly checking up the preceding speakers.

Dr. H. A. West, of Galveston, responded to the toast, "Our Association." This gentleman is the possessor of rare intellect and his countenance discloses it. He is of a serious nature, somewhat stern, but kindness and patience are side by side, with courage and resolve in the gleam of his eye. He spoke of his Kentucky home, and the hospitality of Cuero, contrasting them most favorably. He spoke of the labors at the meeting in Cuero, and said it was a most pleasant surprise to him and other gentlemen present that in an interior town of Texas such striking culture, generosity, talent and ability were to be found, not only in social affairs but among business and professional men, and especially among the physicians. He was delighted with her entertainment and welcome, and more than gratified at the high character of the papers read and speeches made at the meeting. He stated that it was the object of the association to originate a suitable drainage system, to devise, if possible, means of insuring pure food and water, to prevent by quarantine all epidemics of contagious diseases and to prevent imposition on the public by quacks and humbugs. He spoke of the failure to secure legislation along these lines, and said some time a law compelling vaccination would be passed. The doctor displayed great thought, zeal and sincerity in his remarks, as well as indication of much reading. He is a strong man. He concluded his address with a few remarks of his affection for Texas, and especially his Galveston home, closing with the lines from "Lalla Rookh," beautifully appropriate to his sentiments.

W. J. Baker, Esq., in responding to the toast, "The Relation of Medicine to the Law," emphasized the great assistance medicine has rendered the law in the definition and punishment of crime through the investigation of its members, giving the legislatures and courts tests and indicia of mental disease, whereby legal and moral responsibility for acts committed could be intelligently determined. Assuming the intent to do evil was the main feature of all criminal inquiry, medical science has thrown the light of investigation on brain action in its normal and abnormal state, and has defined its features and manifestations that the punishment of insane persons for acts otherwise criminal could be humanely avoided, as well as furnishing safe tests to detect simulated insanity in sane criminals on trial. He congratulated them for the broad discoveries in sanitation, their bold and liberal views and their benefits to the race, but reminded them that his own profession was one of high aims and aspirations, one that had ever struggled for liberty of thought and conscience and the uplifting of humanity, that the two sciences were the greatest and most potent in their impress upon the race, and stood hand in hand in their great efforts, and that each was indebted to the other for that which is best in each.

Dr. J. W. Scott, of Houston, is a practical man, whose face shows experience with men as well as kindness, candor and sound judgment.

The doctor did not speak long, but what he said was to the point. He spoke of the cures discovered lately; the progress on the yellow fever serum, and said that experiments were proceeding that promised most favorably a cure for consumption. He mentioned the great decrease in malaria as due to medical science; he briefly adverted to the wonderful feats in surgery—the removal of the stomach, the vermiform appendix, operations on arteries, and many other successful operations; he seemed imbued with the rational belief that in the course of another decade human life would be prolonged, and the most dreaded diseases eliminated from the human family. He said that a northern man said the south was the most hospitable section of the United States, Texas the most hospitable State in the south, and he would add Cuero was as hospitable as any place in Texas, if not more so.

Dr. R. T. Morris, of Houston, is a handsome young medico about thirty-six years of age. His good looks are backed by a long head, if it is round. He is a cultured gentleman of retiring disposition, with an eye that tells of much reading, accurate judgment, keen observation and a happy disposition towards mankind; he is a magnetic fellow, with a vein of genuine humor. His toast was, "The Ladies." He said that he did not like to speak upon a subject that he did not understand, and that he had tried diligently, and as yet had failed to grasp woman. He said that he entertained ideas exceedingly poetic about the ladies, but there was something unreal about poetry, and that he was afraid these might do them an injustice; that he would say that he had heard that the highest and noblest conceptions of the artist were never transferred to canvas; the sweetest songs are never sung, the grandest thoughts fade as a dream, and are never expressed, and that he hoped that his ideas of woman would be likewise rated, as he felt unable to express them. His fluency of language and happy expression were exceedingly pleasing, and his delicate humor was much enjoyed.

Dr. J. M. Reuss, the patriarch of the physicians of South Texas, the friend of more people than any man present, said that it was intended that Col. D. C. Proctor, the nestor of the Cuero bar, should preside, but that he was unexpectedly called from home. That if Col. Proctor had been present, he would have said that he (Proctor) was an immune, because Dr. Reuss had practiced on him for forty years and had not killed him. The doctor said that he would have replied to this that Col. Proctor was in fact an immune. That he was out walking one evening near the railroad track, and near a convict camp, and about the time the Colonel came in range some of the convicts tried to escape, and the guards shot the Colonel, but that they were like him (Dr. Reuss); they couldn't kill him.

The speeches were all elevating, manly, chaste and instructive and entertaining, with sufficient sarcasm, wit and humor. The toast-master was assailed on all sides for remarks made by him in introduction, or possibly because he was a member of the legislature, but he came up smiling after each round, and was game to the finish; in fact, he was so game a vote of thanks was unanimously given him.

Thus ended a most enjoyable evening, and a most successful meeting of the South Texas Medical Association.—*Cuero Record.*

The following acrostic, written by a lady of the Hospital Society, accompanied the floral presentation to Dr. Joe Reuss on Tuesday night:

Should time bring back the memory of this night,
Ah! may these flowers sweet recollections bring.
Like their sweet charms, thy deeds bring rays of light
On paths once dark that now with gladness sing.
May sweet rewards and blessings cheer thy home,
Each year bring fresh success to our Salome.

